# Prevalence and correlates of cognitive impairment in schizophrenia: a cross-sectional study from a teaching hospital southern Sri Lanka

**DOI:** 10.1186/s12888-022-04368-2

**Published:** 2022-11-17

**Authors:** Praveen Goonathilake, Dileepa Ediriweera, Rumi Ruban, Amila Isuru

**Affiliations:** 1 Mental Health Unit, Teaching Hospital Karapitiya, Galle, Sri Lanka; 2 Health Data Science Unit, Faculty of Medicine, University of Kelaniya, Kelaniya, Sri Lanka; 3 Department of Psychiatry, Faculty of Medicine and Allied Sciences, Rajarata University of Sri Lanka, Mihintale, Sri Lanka

**Keywords:** Schizophrenia, Cognitive symptoms, Correlates, Associated factors, Addenbrooke’s cognitive examination

## Abstract

**Introductions:**

This study assessed the prevalence of cognitive impairment, the degree of impairment in individual cognitive domains and sociodemographic and clinical correlates among patients attending to psychiatry clinics at Teaching Hospital, Karapitiya, Sri Lanka.

**Methods:**

A cross-sectional study was carried out at the psychiatry outpatient clinics of Teaching Hospital, Karapitiya, Sri Lanka. Their cognitive functions were assessed using the culturally validated Sinhala version of Addenbrooke’s Cognitive Examination – III (ACE-III-S). ACE-III-S score below 85.5 was considered as significant cognitive impairment. Linear regression analysis was used to assess the factors associated with cognitive impairment. A *P* value of 0.05 is considered significant.

**Results:**

One hundred forty patients with schizophrenia were assessed. Of this, 125 patients had significant cognitive impairment with a prevalence of 89.3% (95% CI:84.1–94.5). Impairment in each cognitive domain was as follows: 60% in attention, 65.7% in memory, 55% in fluency, 61.4% in language, and 63.6% in visuospatial skills. Impairment was not different between cognitive domains. Advancing age (*P* < 0.001), shorter duration of formal education (*P* = < 0.001), longer duration of illness (*P* = < 0.001) and not having a full-time employment (*P* = 0.020) showed a positive association with cognitive impairment.

**Conclusions:**

Nine out of ten patients with schizophrenia experienced significant cognitive impairment. Patients showed more than 50% impairment in all cognitive domains. The cognitive domains did not show disproportionate impairment. This study highlights the importance of introducing routine cognitive assessment protocols in patients with schizophrenia.

## Background

Schizophrenia is a serious mental disorder affecting around 20 million individuals worldwide [1]. It is the third leading cause of morbidity among people aged between 15 and 44 in the world [2]. Antipsychotic medications improve psychotic symptoms manifested by the disease. However, the degree of improvement in terms of functional level among patients with schizophrenia remains unsatisfactory up to now [3]. Research evidence has consistently shown that cognitive impairment predicts the poor functional level of patients with schizophrenia [4]. Cognitive deficits appear to predate the onset of positive symptoms of schizophrenia and remain relatively stable throughout the illness rather than progressively declining [5].

A meta-analysis of 204 studies by Heinrichs and Zakzanis in 1998 found that cognitive deficits are common in schizophrenia [6]. Studies from the USA, Nepal and Thailand have reported the prevalence of significant cognitive impairment as 81.9, 86.7 and 81.3% respectively [7, 8]. While working memory, verbal fluency, verbal learning and memory, and executive functioning are considered the most widely affected cognitive domains [9], some studies have indicated that verbal memory and executive functions are more severely affected than other domains [9]. Potential correlates of cognitive dysfunction have been investigated widely around the world. Out of them, male gender, earlier age of onset, presence of metabolic risk factors, a higher dose of antipsychotics, use of anticholinergic medications, and smoking have shown evidence of association with more severe cognitive impairment [10–12] [13, 14].

The high prevalence of cognitive impairment across multiple domains suggests that cognitive dysfunction is an integral part of schizophrenia. Number of studies published since the early 1990s have shown that cognitive deficits are the best predictor of functional outcome of patients with schizophrenia [15, 16]. Although medications do not appear to improve them, the application of novel therapeutic options such as cognitive remediation therapy has shown to be effective [17]. Therefore, early identification of cognitive issues has become vital in managing patients.

Although cognitive dysfunction in schizophrenia has been widely studied in Western countries, data related to Asian populations are limited. The knowledge gap created by the limited data related to cognitive deficits in patients with schizophrenia in the region justifies our study. The study aimed to estimate the prevalence of cognitive impairment among patients with schizophrenia, assess the degree of impairment in individual cognitive domains, and to identify the sociodemographic and clinical factors contributing to it.

## Methods

### Study population and sample selection

The study population was the patients diagnosed with schizophrenia who attend the psychiatry outpatient clinics at the Teaching Hospital, Karapitiya, which is the largest teaching hospital in Southern Sri Lanka. Diagnosis of schizophrenia was made by a consultant psychiatrist based on ICD 10 criteria. Two general adult psychiatry clinics run by the Hospital Psychiatry Unit and the University Psychiatry Unit were selected. Patients who presented with acute symptoms of schizophrenia, co-morbid intellectual disability and/or had electroconvulsive therapy, head injury or intracranial infection during the last 3 months or less than 18 years of age were excluded from the study since these circumstances were likely to interfere with reliable measurement of their cognitive performance. The study was carried out from May 2020 to June 2021.

We identified 203 patients eligible for the study and 34 patients refused to give informed consent. Therefore, we did not have an adequate sample to randomize the study sample. Hence, we interviewed all the patients until the sample size is achieved.

### Sample size

A sample size of 138 was determined assuming a 90% of expected prevalence of cognitive impairment among patients with schizophrenia (p), a margin of error of 5% (d) and a confidence level of 95% (Z) [18].

### Data collection

All the patients enrolled on the study were interviewed by the principal investigator. This interview was composed of three main sections. The initial section was dedicated to obtaining informed consent. This was followed by the completion of an interviewer-administered questionnaire designed to collect data on socio-demographic details, past psychiatric history, family history of psychiatric illness, medical co-morbidities, current medications, and substance history of the participant. In addition to the verbal information provided by the patients, their clinic records, diagnosis cards and if present, collateral information provided by their family members were used to fill this questionnaire. In the final section of the interview, the patient’s cognitive functions were assessed using the culturally validated Sinhala version of Addenbrooke’s Cognitive Examination version III (ACE-III-S) [19]. ACE-III is a test battery principally used to measure the cognitive performance of individuals suspected of having dementia [19]. However, published research showed ACE III is sensitive to screening cognitive symptoms of schizophrenia [20]. It assesses five cognitive domains with a specified maximum score for each of them: attention (maximum 18), memory (maximum 26), fluency (maximum 14), language (maximum 26), and visuospatial abilities (maximum 16). The total score is calculated out of 100, which is the maximum possible score of this scale. Brief Psychiatry Rating Scale (BPRS) was used to rule out patients with acute symptoms of schizophrenia.

Anticholinergic Cognitive Burden (ACB) scale was used to measure the cumulative anticholinergic effect of medications for each patient. Based on the degree of anticholinergic effect, this scale gives a score between 0 and 3 for each medication an individual is on [21].

### Outcome measures

The total score of ACE-III-S was the primary outcome measure of the study. The patients who scored less than 85.5 were categorized as showing significant cognitive impairment [19]. The secondary outcome measures were the subtotal scores of five cognitive domains measured by ACE-III-S. The patients who scored below the lower limits of normative values (mean – 2SD) generated from the ACE-III-S validation study [19] were categorized as having deficits in respective cognitive domains (i. e. cut-off for attention: 15, memory: 20, verbal fluency: 7, language: 22, visuospatial abilities: 14).

### Data analysis

Descriptive data were analysed and tabulated to present the study sample characteristics. The proportion of patients who scored below the lower limits of normative values for each cognitive domain in ACE-III-S was calculated. Poisson log-linear analysis was performed to compare the statistical difference between these percentages. A simple linear regression analyses were carried out to identify the independent socio-demographic and clinical variables that are associated with the total ACE-III-S score of the patients. Variables examined in the regression analysis were age, gender, handedness, years of formal education, relationship status, full-time employment, age of onset, duration of illness, number of admissions to psychiatry units, history of ECT, use of combined antipsychotics, use of clozapine, use of long-acting injectables (LAIs), the dose of antipsychotics (in eq-CPZ)), ACB score, metabolic risk factors, history of head injury before the onset of illness, history of substance use, and family history of mental illness. The IBM® SPSS® Version 22 and R programming language version 4.1.1. were used to do the statistical analysis. A *P* value of < 0.05 was considered as significant.

### Ethical consideration

The ethical approval for the study was obtained from the Ethics Review Committee, Faculty of Medicine, University of Ruhuna.

## Results

### Characteristics of the sample

Out of 140 patients enrolled in the study, 73 (52.1%) were males. The age ranged from 21 to 76 and the mean age of 46.8 years (SD = 12.2). The mean duration of formal education received by the patients was 10.2 years (SD = 3.2).

Table 1 summarizes the demographic and illness-related characteristics of the patients.

**Table 1:** Characteristics of the Sample

	Overall (*n* = 140)		Male (*n* = 73)		Female (*n* = 67)	
Mean age (SD)	46.8 (12.2)		46.0 (12.4)		47.8 (12.0)	
Civil status						
Single	77	(55.0%)	47	(64.4%)	30	(44.8%)
Married	44	(31.4%)	19	(26.0%)	25	(37.3%)
Separated	06	(4.3%)	03	(4.1%)	03	(4.5%)
Divorced	08	(5.7%)	03	(4.1%)	05	(7.5%)
Widowed	05	(3.6%)	01	(1.4%)	04	(6.0%)
Level of education						
No school education	02	(1.4%)	01	(1.4%)	01	(1.5%)
Grade 1–5	15	(10.7%)	08	(11.0%)	07	(10.4%)
Grade 6–11	70	(50.0%)	39	(53.4%)	31	(46.3%)
O/L passed	14	(10.0%)	07	(9.6%)	07	(10.4%)
Grade 12–13	17	(12.1%)	10	(13.7%)	07	(10.4%)
A/L passed	18	(12.9%)	05	(6.8%)	13	(19.4%)
University education	03	(2.1%)	02	(2.7%)	01	(1.5%)
Postgraduate education	01	(0.7%)	01	(1.4%)	–	–
Occupational category						
Unemployed	69	(49.3%)	37	(50.7%)	32	(47.8%)
Employed - full time	30	(21.4%)	22	(30.1%)	08	(11.9%)
Homemaker	21	(15.0%)	–	–	21	(31.3%)
Employed - part time	10	(7.1%)	08	(11.0%)	02	(3.0%)
Retired	07	(5.0%)	03	(4.1%)	04	(6.0%)
Self-employed	03	(2.1%)	03	(4.1%)	–	–
Medical co-morbidities						
Diabetes mellitus	17	(12.1%)	10	(13.7%)	07	(10.4%)
Hypertension	12	(8.6%)	06	(8.2%)	06	(9.0%)
Hypercholesteraemia	06	(4.3%)	05	(6.8%)	01	(1.5%)
Epilepsy	02	(1.4%)	01	(1.4%)	01	(1.5%)
Special treatment modalities						
ECT at some point in the course of illness	73	(51.2%)	41	(56.2%)	32	(47.8%)
Clozapine	57	(40.7%)	34	(46.6%)	23	(34.3%)
Combined antipsychotics	50	(35.7%)	31	(42.5%)	19	(28.4%)
LAIs	18	(12.9%)	09	(12.3%)	09	(13.4%)

The mean age of onset of the illness was 27.3 years (SD = 9.8) and the mean duration of illness was 19.3 years (SD = 10.6). The total number of admissions to psychiatric wards since the onset of illness was less than 5 in 75.7% (106) patients. A family history of psychiatric disorder was present in 41 (29.3%) patients. A history of head injury before the onset of illness was reported by 7 (5%) patients.

When the antipsychotic doses were converted to chlorpromazine equivalents (Eq-CPZ) (16), the dose of antipsychotics taken by the patients ranged from 50 to 1350 mg of chlorpromazine with a mean value of 356 mg (SD = 216). Two or more antipsychotics (combined antipsychotics) were received by 50 (35.7%) patients, while 18 (12.9%) of them were on LAIs. Number of patients treated with clozapine was 57 (40.7%). The mean ACB score was 4.61 (SD = 1.9).

### The extent of cognitive impairment

The total ACE-III score in the sample ranged from 21 to 95 with a mean value of 67.9 (SD =14.7). When the cut-off score of 85.5 was considered, 125 (89.3%; 95% CI:84.1–94.5) patients had significant cognitive impairment.

When the scores of individual cognitive domains were compared with the lower limit of normative values (−2SD) obtained from the ACE-III-S validation study, it was found that the mean scores of all five domains were below the lower limit (Table 2). Furthermore, the percentage of patients who scored below the lower limit in each cognitive domain ranged from 55.0 to 65.7% with memory being the most widely affected domain. However, there was no statistically significant difference between the domains.

**Table 2:** Comparison of Subtotal Scores of Individual Cognitive Domains with Normative Values

Cognitive domain	Maximum possible marks	Lower limit of normative values	Sample mean (SD)		Percentage below lower limit	Significance (P value)^a^
Attention	18	15	14.01	(2.62)	60.0%	0.704
Memory	26	20	16.23	(5.35)	65.7%	0.824
Fluency	14	07	6.18	(2.77)	55.0%	0.352
Language	26	22	19.46	(4.46)	61.4%	0.821
Visuospatial	16	14	12.01	(2.91)	63.6%	-^b^

^a^Significance (*P* value) generated by the Poisson log-linear analysis

^b^Visuospatial skills domain was taken as the reference category in Poisson log-linear analysis

### Explorative analysis for correlates

**Fig. 1:**
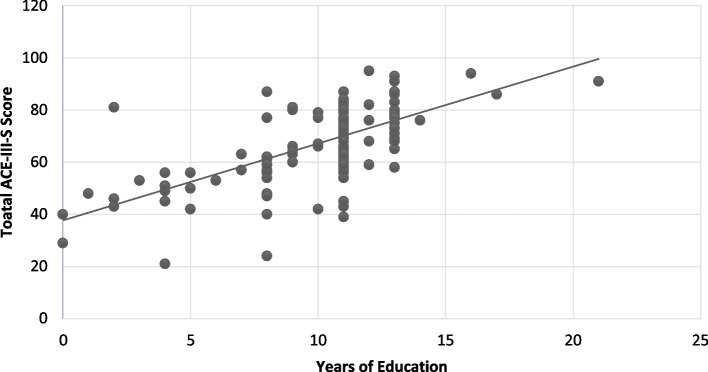
Correlation between Years of Education and Total ACE-III-Score

**Fig. 2:**
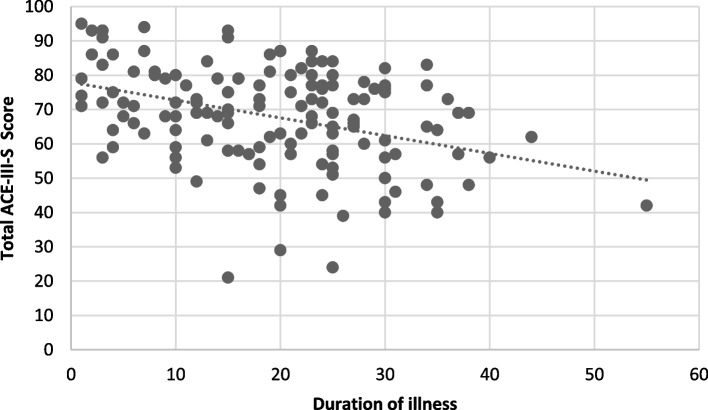
Correlation between Duration of Illness and Total ACE-III-S Score

Table 3 illustrates the findings of individual variable analysis. The analysis showed advancing age, lesser years of formal education, longer duration of illness and not having full-time employment were significantly associated with cognitive impairment (Figs. 1 and 2).

**Table 3:** Individual variable analysis

Independent Variable	N	Beta	95% CI^*1*^	*p*-value
Age	140	−0.52	−0.70, −.33	< 0.001
Gender	140	1.0	−4.0, 5.9	0.7
Handedness	140	0.05	−7.7, 7.8	> 0.9
Years of formal education	140	2.9	2.4, 3.5	< 0.001
Relationship status	140	−4.0	−9.2, 1.3	0.14
Fulltime employment	140	7.1	1.1, 13	0.020
Metabolic risk factors	140	−0.11	−6.1, 5.9	> 0.9
Age of onset	140	−0.21	−0.46, 0.04	0.10
Duration of illness	140	−0.518	−0.73, − 0.30	< 0.001
Number of admissions	140	−1.7	−7.8, 4.4	0.6
History of ECT	140	−3.3	−8.2, 1.7	0.2
History of head injury	140	−6.2	−18, 5.1	0.3
Dose of antipsychotics	140	−0.01	−0.02, 0.00	0.10
Polypharmacy	140	−2.6	−7.7, 2.6	0.3
Clozapine	140	2.4	−2.6, 7.4	0.3
LAIs	140	−5.6	−13, 1.8	0.14
ACB score	140	−0.69	−1.9, 0.56	0.3
Substance use	140	−5.3	−11, 0.74	0.085
Family history	140	5.1	−0.29, 10	0.064

## Discussion

This study showed the prevalence of cognitive impairment among patients with schizophrenia was 89.3% which was comparable to the estimates of most other studies [20, 22]. While the prevalence rates of many studies ranged between 80 and 90% [22], a study from the USA reported the prevalence as 98% [23]. Published data regarding this topic in Asian countries is limited, a study from Nepal found the prevalence of working memory impairment in a sample of Nepalese antipsychotic-naive patients with schizophrenia to be 86.7% [22]. A descriptive study from Thailand showed that 81.3% of the study sample had significant cognitive impairment when measured with Montreal Cognitive Assessment Tool (MoCA-T) [8]. However, in both these studies, the sample size was small (30 and 81 respectively), and the tools used to assess cognitive functions appear to be less sensitive to capture the full range of cognitive impairment in patients with schizophrenia compared to our study. A multicenter study, conducted in Japan with a representative sample of patients with schizophrenia, estimated the cognitive decline after the onset of schizophrenia with the Adult Wechsler Adult Intelligence Scale III (WAIS-III) was 70% [24]. Another research conducted in China showed that the first episode and patients with chronic schizophrenia scored lower compared to the normal control [21].

Studies from Western countries have reported an equally high prevalence [25]. Therefore, the findings of our study further strengthen the notion that cognitive impairment is an integral part of the illness [26, 27].

All five cognitive domains assessed in this study (attention, memory, fluency, language, and visuospatial skills) showed lower mean scores than the lower limit of normative values. The fact that the cognitive domains are equally impaired, or some domains are disproportionately affected in schizophrenia is a matter of debate in the scientific community. For example, Bilder et al., 2002 reported that verbal memory and executive functions are more severely affected in schizophrenia than other cognitive domains while Blanchard JJ, Neale JM. 1994 failed to detect a disproportionate impairment between domains when a group of 28 patients with schizophrenia were compared with matched normal subjects using a battery of neuropsychological tests [28]. In our study sample, memory was the most widely affected domain with 65.7% of the patients scoring below the lower limit whereas fluency was the least affected with 55.0% of patients scoring below the lower limit. However, the results of the Poisson log-linear analysis concluded that the differences noted between the domains in terms of the percentage of affected patients were not statistically significant. This supports the view that cognitive impairment in schizophrenia is more likely to be generalized rather than disproportionate across different cognitive domains.

Longer years of education showed a positive association with the cognitive performance in our study. This positive association is understandable as it is well-recognized that people with better education attainment generally perform better in cognitive function tests [11]. In addition, it is speculated that greater educational achievements bring about more favourable life conditions through better occupational status and low health risks. This suggests that the level of education might also have indirect effects on cognitive performance in later life [29].

This study also showed that full-time employment was associated with higher ACE 111 scores. Cognitive impairment is a predictor of poor workplace functioning [30].

Advancing age is a risk factor for cognitive impairment [31]. The finding of this research on advancing age associated with cognitive impairment is compatible with the research evidence that aging is a risk factor for cognitive impairment in patients with schizophrenia [32].

This study also identified the duration of illness is significantly associated with the cognitive performance of the participants; the duration of illness exhibited a negative association. The consensus regarding the progress of cognitive dysfunction in schizophrenia is that it is static rather than progressive [33–35]. However, it should be noted that this view has been challenged by some clinicians who have recognized that individual patients show some degree of cognitive decline throughout illness [36]. A 10-year follow-up study showed a significantly larger decline in IQ, memory, verbal learning, immediate recall, delayed recall, and vocabulary in patients with schizophrenia as compared to other psychoses [37].

Given the high prevalence and the impact of outcome of patients with schizophrenia, early identification of cognitive dysfunction is becoming crucial in the management of schizophrenia. Currently, no adequate attention is paid to the cognitive functions of patients during routine assessments in most psychiatry settings in Sri Lanka. Therapeutic nihilism surrounding this issue could be an important factor behind this lack of interest. Early commencement of cognitive rehabilitation programs could play a vital role in minimizing the impact.

### Limitations

We identified several limitations of this study. Being a cross-sectional study, the findings of this study cannot be used to establish the direction of causation. Since participants were recruited from the outpatient clinics, the category of patients with more severe negative symptoms, poorer functional level, poor medication adherence, and less likelihood of regular clinic attendance may not have had an adequate representation in the sample. Data on medications represent only current medications rather than a longitudinal medication history. Clinical assessment and cognitive assessment were performed by one investigator which may cause a bias in the assessment of cognitive functions. The prevalence of cognitive symptoms of schizophrenia cannot be generalized to the Asian population as the sample was selected from patients attending to mental health clinic of a teaching hospital in southern Sri Lanka. This study did not compare the prevalence of cognitive symptoms of patients with schizophrenia with a healthy control group. This limits the ability to interpret results. We did not perform semi-structured interviews to supplement neurocognitive assessments in this study which may limit the real-life assessment of neurocognitive deficits in schizophrenia.

## Conclusions

The findings of this study confirmed that cognitive dysfunction is widespread among patients with schizophrenia with many cognitive domains are affected to a significant degree. The prevalence is comparable to observations made in both South Asian and Western countries. These results emphasize the importance of paying closer attention to this crucial aspect of the illness preferably by introducing protocols to implement routine cognitive assessments during initial patient assessments and subsequent reviews.

## Data Availability

The data sets used and analysed during the current study are available from the corresponding author on reasonable request.

## Abbreviations

ACB: Anticholinergic Cognitive Burden
ACE-III: Addenbrooke’s Cognitive Examination III
ACE-III-S: Addenbrooke’s Cognitive Examination - Sinhala Version
BPRS: Brief Psychiatric Rating Scale
CATIE: The Clinical Antipsychotic Trials of Intervention effectiveness
DSM-5: Diagnostic and Statistical Manual of Mental Disorders, 5th Edition
ECT: Electroconvulsive Therapy
Eq-CPZ: Chlorpromazine Equivalents
FDA: Food and Drug Administration (USA)
GDS: Global Deficit Score
ICD-10: The International Classification of Diseases, 10th Revision
IM: Intramuscular
IQ: Intelligence Quotient
LAI: Long Acting Injectable
MOCA-T: Montreal Cognitive Assessment - Thai version
